# Political regime, data transparency, and COVID-19 death cases

**DOI:** 10.1016/j.ssmph.2021.100832

**Published:** 2021-06-12

**Authors:** Susumu Annaka

**Affiliations:** Waseda Institute for Advanced Study, Waseda University, 1-104 Totsukamachi, Shinjuku-ku, Tokyo, 169-8050, Japan

**Keywords:** COVID-19, Political regime, Data transparency, Data manipulation

## Abstract

The COVID-19—the worst pandemic since the Spanish flu—has dramatically changed the world, with a significant number of people suffering from and dying of the disease. Some scholars argue that democratic governments are disadvantaged in coping with the current pandemic mainly because they cannot intervene in their citizens' lives as aggressively as their authoritarian counterparts. Other scholars, however, suggest that possible data manipulation may account for the apparent advantage of authoritarian countries. Taking such a possibility seriously, this paper analyzes the relationship between political regimes, data transparency, and COVID-19 deaths using cross-national data for over 108 countries, obtained from Worldometer COVID-19 Data, Polity V Project, Variety of Democracy (V-Dem) Project, HRV Transparency Project among other sources. Regression analyses indicate that authoritarian countries do not necessarily tend to have fewer COVID-19 deaths than their democratic counterparts after controlling for other factors, especially data transparency. The transparency variable itself, on the other hand, is positively correlated with the number of death cases more consistently (*P* <0.05). Overall, the estimation results point to the possible data manipulation, not the nature of regime characteristics itself, as a more significant source for the seemingly low casualty rates in authoritarian countries.

## Highlights

• Authoritarian countries do not necessarily tend to have fewer COVID-19 deaths than their democratic counterparts.
• Data transparency is positively correlated with the number of death cases more consistently.
• The findings point to the possibility of data manipulation as a plausible source for fewer deaths in authoritarian governments.

## 1 Introduction

The number of COVID-19 deaths is reported to have exceeded 2.8 million across the world (as of April 1, 2021). While policies taken against the pandemic differ from one place to another, there are two divergent views on the effect that different political regimes may have in preserving national public health. Some scholars argue, on the one hand, that democratic governments are disadvantaged in combatting the spread of formidable diseases, like COVID-19, because the respect for individual rights and freedom precludes them from taking aggressive or drastic measures which their authoritarian counterparts could adopt (Alsan et al., 2020; Norheim et al., 2020; Thomson & Ip, 2020). A series of recently published papers show that democratic countries suffer from more COVID-19 deaths than authoritarian states (Cepaluni et al., 2020; Cheibub et al., 2020; Frey et al., 2020). Cassan and Steenvoort (2020) called this line of literature the “efficient autocracy” view.

For those scholars who uphold the legitimacy and core values of modern democracy, on the other hand, the claim that nondemocracies are better at coping with the pandemic remains unconvincing. It has been reported, for example, that some authoritarian leaders, like Alexander Lukashenko in Belarus, blatantly underestimated the risk of the new virus and failed to adopt countermeasures in a timely and appropriate fashion. The reported low casualty rates in these countries (Karáth, 2020), therefore, might not be attributable to decisive actions and interventions taken by their governments. Besides, it is difficult to imagine, more fundamentally, that authoritarian states' healthcare systems would work better than those of democratic countries. The conventional wisdom in the relevant literature suggests the opposite indeed, as scholars have found that people in democratic countries are likely to have better health than their authoritarian counterparts (Gerring et al., 2020; Kavanagh & Singh, 2020; Wang et al., 2019).

One possible reason that may account for the apparent authoritarian advantage is that authoritarian countries manipulate death data. Cassan and Steenvoort (2020) called this line of reasoning the “biasing autocracy” view. Kapoor et al. (2020), for example, analyzed the moving average of the reported number of deaths in authoritarian countries, revealing that the published data are likely to be unnaturally produced. Adiguzel et al. (2020) also reported a similar result for the governmental statistics of digit-based tests.

Building upon these studies that take the possible data manipulation seriously, this paper explores, by utilizing cross-national data, whether the apparent authoritarian advantage is truly attributable to the regime characteristics. The estimation results indicate that political regime variables are not associated with the number of death cases after controlling for other factors, especially data transparency. The transparency variable itself, on the other hand, is positively correlated with the number of death cases more consistently (*P* <0.05). The measurement of data transparency may, of course, correlate with other governmental characteristics that contribute to the varying death rates. The above results hold, however, even after including the variable that measures bureaucratic capacity or governmental effectiveness. Thus, overall, the results of this paper reject the notion of “efficient autocracy,” pointing to the possible data manipulation as a more significant source for the seemingly low casualty rates in authoritarian countries.

## 2 Data

### 2.1 Political regime

**Fig. 1:**
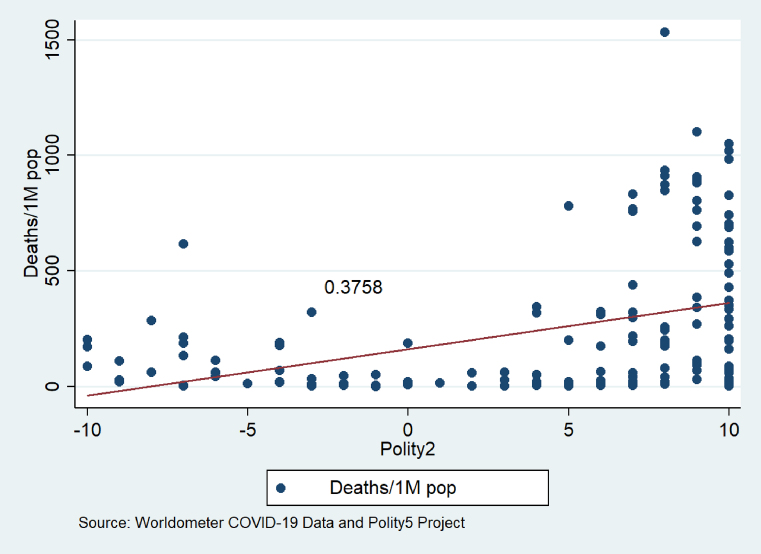
Relationship between Polity2 and the number of COVID-19 deaths.

Fig. 1 plots the total number of COVID-19 deaths per 1 million (as of December 12, 2020) on the vertical axis, as reported by Worldometer COVID-19 Data (Worldometer COVID-19 Data, 2020), and the level of Polity2 in 2018 (latest) on the horizontal axis from Polity V Project (Marshall et al., 2020). The latter codes democracy levels from -10 (most autocratic) to 10 (most democratic). This figure appears to support, at least to some extent, the so-called “efficient autocracy” view. The correlation coefficient between the two variables is 0.3758 (*P* <0.001). Note seventeen countries in the data seem to report zero death by December 2020, though most of them are small countries, and zero cannot be distinguished from missing values according to the data source. When assigning zero for the missing values, the results in the regression analyses below do not change. Note also that countries with very few deaths (for example, under ten) are not visually distinguished from those with zero death on the graphs.

**Fig. 2:**
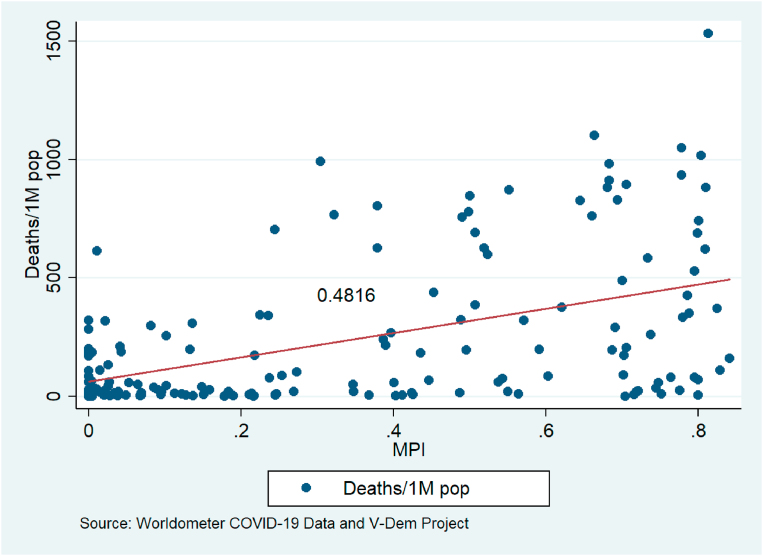
Relationship between MPI and the number of COVID-19 deaths.

Using an alternative measure of the political regime makes the relationship more apparent. Fig. 2 shows the relationship by using the Multiplicative Polyarchy Index (MPI) in 2019 (latest) from Variety of Democracy (V-Dem) Project (Coppedge et al., 2020). This variable is created by multiplying the five core components of electoral democracy: freedom of association (v2x_frassoc), clean elections (v2x_frefair), freedom of expression (v2x_freexp), elected officials (v2x_elecoff) and suffrage (v2x_suffr), and codes democracy levels from low to high (0–1) (Coppedge et al., 2020). The correlation coefficient between these two variables is 0.4816 (*P* <0.001). These moderate, positive relationships appear to support the argument that democratic governments are disadvantaged in coping with the current pandemic.

Are these relationships accurate? A systematic cross-national analysis yields “no” as the answer by taking the possibility of data manipulation seriously, as shown below.

### 2.2 Data transparency

As suggested by the alternative view, namely “biassing autocracy,” it is possible that some authoritarian governments manipulate death data to overstate their successes in combatting COVID 19. Some studies have already highlighted this possibility through incisive but purely statistical inquiries (Adiguzel et al., 2020; Kapoor et al., 2020). This paper turns instead to HRV Transparency Index (Hollyer et al., 2014) as a substantive independent variable to capture the problems of data credibility across countries. HRV Transparency Project creates this index based on the WDI data regarding the missingness/non-missingness in the WDI data to estimate the government's willingness to disclose its country's internal affairs. This index can be a proxy for data transparency or data manipulation.

**Fig. 3:**
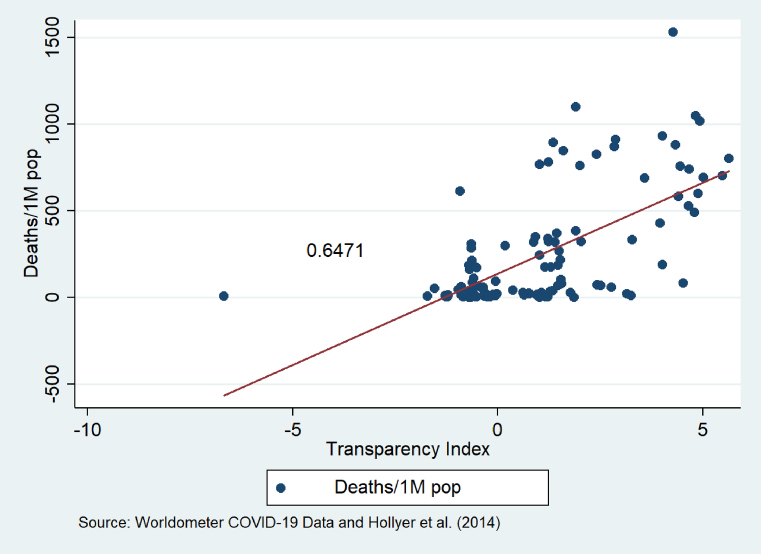
Relationship between transparency index and the number of COVID-19 deaths.

Fig. 3 shows the relationship between HRV Transparency Index in 2010 (latest) and the number of COVID-19 deaths. The correlation coefficient between the two variables is 0.6471 (*P* <0.001). This positive relationship is stronger than the relationships reported between the casualty rates and the two political regime variables, implying that countries that disclose more reliable data tend to report more COVID-19 deaths. Meanwhile, the correlation between HRV Transparency Index and Polity2 is 0.4045, and MPI 0.5455 (*P* <0.001); these results are consistent with the earlier finding reported by Hollyer et al. (2014). Given these relationships, the suspicion that authoritarian countries tend to report data in their favor remains. This tendency may lead to the apparent advantages of authoritarian governments in the current COVID 19 related deaths. In the following section, this paper analyzes this possibility using a statistical method.

## 3 Methods

This section advances a cross-national analysis of the relationship between political regimes, data transparency, and COVID-19 deaths. It estimates the following specification:

$$COVID\;death_{i}=\alpha +\beta_{1}Democracy_{i}+\beta_{2}Transparency_{i}+\beta {'}_{3}X_{i}+\varepsilon_{i}$$

*COVID death* is the total number of COVID-19 deaths per 1 million (as of December 12, 2020). *Democracy* indicates Polity2 Score or MPI, and *Transparency* represents HRV Transparency Index. X is a vector of controls. ε is an error term. *i* represents each country.

The total number of deaths is obtained from Worldometer COVID-19 data. Daily data available elsewhere cannot be utilized for the analysis because almost all other covariates necessary to be included in the analysis, such as economic and demographic measures, are yearly data. This study constructs cross-national data on 108 countries, obtaining political regime variables from Polity V Project and Variety of Democracy (V-Dem) Project. HRV Transparency Index is taken from Hollyer et al. (2014) and included in the analysis. Control variables, namely Government Effectiveness, GDP per capita, population density, and population ratio age 65 and above, are taken from the World Bank. For all variables, the latest available yearly data are used. Government Effectiveness is a variable that denotes the general administrative capacity, such as bureaucratic competence and accountability for public services; it can thus be expected that the higher this score, the better the government can cope with the pandemic. Stringency Index, which captures the level of government intervention in citizens' daily lives, is obtained from Hale et al. (2020) and averaged during the days since the first positive case was reported. Also included as control variables are both the latitude and longitude obtained from Johns Hopkins University (2020), which capture geographic characteristics, such as humidity, cultural factors, such as high awareness of mask usage and preventive behavior affecting the severity of COVID-19 deaths, as well as any remaining regionally specific effects. The total number of pandemic-related tests is obtained from Worldometer COVID-19 data. Finally, both the days since the first confirmed case and the quadratic term of them are included to capture linear and non-linear trends of the infection. The inclusion of these variables worsens the Variance Inflation Factor (VIF), but the results do not significantly change compared to the models without them.

For estimation, log transformation of the dependent variables for OLS and Poisson regression with robust standard errors are applied, considering the dependent variable's skewed distribution. The control variables (except for Government Effectiveness, Stringency Index, latitude, longitude, and days since the first confirmed case) are logged because of their skewed distributions. Model goodness of fit is principally assessed by the Akaike information criterion (AIC) and the Bayesian information criterion (BIC) based on Lindsey (2014) and Gluzmann et al. (2015). Note, however, the variables central to this study, such as the political regime variables, are included for the estimations, regardless of AIC and BIC assessment. The descriptive statistics are presented in Appendix A. HRV Transparency Index yields fewer observations than other variables because, as annotated by Hollyer et al. (2014), it “exclude[s] any country that did not exist for the entirety of the 1980–2010,” “modern countries that are formed by the union of preexisting states during the 1980–2010 period—that is, Germany and Yemen,” and “all micro-states” and, hence, “only include[s] states that maintained a population of 500,000 or more throughout the 1980–2010 period” (419). Such a limitation notwithstanding, this variable is crucial to the study and is thus retained. All the countries analyzed are listed in Appendix B.

## 4 Results

Table 1 shows the regression results for the determinants of the death cases. Models 1 and 2 analyze the relationship between Polity2 from the Polity Project and death cases. Models 3 and 4 analyze the relationship between MPI from V-Dem Project and death cases. Models 1 and 3 take logs of the dependent variables and run linear regressions. Models 2 and 4 run Poisson regressions for models 1 and 3, respectively. These results do not confirm the association found in Fig. 1, Fig. 2 above. After controlling for various factors, in other words, the results rather show that authoritarian countries do not tend to have fewer COVID-19 deaths. Neither Polity2 nor MPI is statistically significant at the conventional level in any of the models presented. Throughout the entire analysis, Transparency Index is always estimated to be positively correlated with the number of deaths at the conventional statistical level (*P* <0.05). Transparency Index is far more robustly associated with reported COVID-19 deaths than the political regime variables. The increase in Transparency Index (which ranges from -1.54 [least transparent] to 5.64 [most transparent]) from the 25th to 75th percentile leads to report about 36 percent more deaths per 1 million people. This is calculated based on Martin et al. (2012) as exp (2.83 × 0.109) -1=about 0.36=about 36%, where 2.83 is the interquartile range in the index, and 0.109 is the coefficient of the index in Model 1 of Table 1.

**Table 1:** Determinants of death cases.

VARIABLES	(1)	(2)	(3)	(4)
	OLS	Poisson	OLS	Poisson
	Deaths cases per 1M (log)	Deaths cases per 1M	Deaths cases per 1M (log)	Deaths cases per 1M
Polity2	0.00878	-0.00460		
	(0.0146)	(0.0146)		
MPI			0.685	0.281
			(0.419)	(0.299)
**Transparency Index**	0.109**	0.143***	0.114**	0.145***
	**(0.0486)**	**(0.0370)**	**(0.0476)**	**(0.0368)**
Government Effectiveness	-0.0437	-0.176**	-0.126	-0.197**
	(0.147)	(0.0845)	(0.155)	(0.0842)
Stringency Index	0.0227***	0.0102	0.0246***	0.00983
	(0.00791)	(0.00701)	(0.00785)	(0.00697)
GDP per capita (log)	0.0217	0.0518	0.000174	0.0343
	(0.0897)	(0.0807)	(0.0960)	(0.0907)
Population Density (log)	-0.171**	-0.0854**	-0.170***	-0.0940**
	(0.0676)	(0.0405)	(0.0631)	(0.0391)
Age 65 and above (ratio)	0.336**	0.284*	0.246*	0.182
	(0.145)	(0.146)	(0.141)	(0.122)
Confirmed cases per 1M (log)	0.901***	0.888***	0.886***	0.895***
	(0.0779)	(0.0884)	(0.0738)	(0.0923)
Tests per 1M (log)	-0.236**	-0.328***	-0.203*	-0.314***
	(0.105)	(0.0866)	(0.106)	(0.0879)
Latitude	0.00414	0.000755	0.00517	0.00154
	(0.00366)	(0.00216)	(0.00367)	(0.00222)
Longitude	-0.00192*	-0.00367***	-0.00123	-0.00318***
	(0.00114)	(0.00124)	(0.00123)	(0.00121)
Days since the first confirmed case	0.0447	0.280**	0.0417	0.270**
	(0.0740)	(0.131)	(0.0726)	(0.128)
Days since the first confirmed case^2	-7.01e-05	-0.000446**	-6.42e-05	-0.000430**
	(0.000126)	(0.000219)	(0.000123)	(0.000213)
Constant	-9.381	-44.23**	-9.235	-42.57**
	(10.77)	(19.57)	(10.57)	(19.13)
Observations	108	108	108	108
Adjusted (or Pseudo) R-squared	0.9133	0.915	0.9162	0.9158
AIC	214.034	3652.128	210.3651	3618.956
BIC	251.5838	3689.678	247.9149	3656.506

Robust standard errors in parentheses.

*** p<0.01, ** p<0.05, * p<0.1.

Government effectiveness is negatively associated with deaths, though not robustly, but significantly in Models 2 and 4 at the conventional level. These results indicate, as expected, that the high administrative capacities do contribute to lowering the rates of casualties. More importantly, perhaps, these results further clarify the significance of the effect captured by Transparency Index. It is possible that this data transparency variable, if it stood alone in the model, could have represented effects beyond data manipulation. While it is difficult to control for all the unobservable effects that correlate with this variable, the Government Effectiveness variable captures at least some of these effects. The results in Table 1 show that this inclusion does not affect the significance of Transparency Index nor the insignificance of the political regime variables.

The analysis thus far, of course, does not rule out the possibility that factors other than data transparency may also be powerful enough to relinquish the spurious effect of regime types. To explore this possibility, further estimations of models, excluding Transparency Index but with a variety of combinations of other control variables, have been conducted. This additional investigation (not reported) has identified one demographic variable, the population ratio over age 65, as another powerful factor in that its inclusion into the estimation models reduces the significance of Polity2 and MPI. This, however, hardly spoils the importance of the above finding regarding the effect of Transparency Index. Given the nature of the pandemic, it is not at all surprising that the elderly in the population is more vulnerable to the disease, and the countries' age structure is strongly correlated with the national death rates (Liang et al., 2020). Furthermore, it is well established that, on average, democratic countries tend to have more elderly population; in the dataset used for this study, the correlation coefficients between the variable for the population ratio over the age of 65 and the regime-type variables are 0.5010 (*P* <0.001) for the Polity and 0.6538 (*P* <0.001) for MPI respectively. Finally, while Transparancy Index and the population ratio over the age of 65 both expose the spurious effects of political regimes on the counts of COVID-related death, a simple and direct test confirms that the former is more salient than the latter. As presented in Appendix C, the results from the estimation models that include these two variables and political regimes show the statistically significant effect of the Transparency variable, while the demographic variable turns out to be no longer significant.

Overall, the findings presented in this paper point to the possible data manipulation, not the nature of regime characteristics itself, as a more significant source for the seeming low casualty rates in authoritarian countries.

## 5 Discussion

The results above support the “biasing autocracy” view rather than the “efficient autocracy” one. Cassan and Steenvoort (2020) also have reported the null effect of the political regime variables after including various controls. Kapoor et al. (2020) and Adiguzel et al. (2020) detected through statistical inquiries the possibility that some authoritarian countries might have manipulated COVID-19 death data. The findings presented in the previous section further support this view, based on systematic cross-national analyses of the relationship between political regimes, data transparency, and COVID-19 deaths.

Apart from the salience of data transparency, the findings presented above highlight two more important patterns worthy of discussion. First, they confirm robustly the effect associated with the frequency of tests conducted. In fact, this is the only factor, except for population density, that is consistently negatively correlated with the number of deaths (*P* <0.1). This result, consistent with Liang et al. (2020), which stressed the importance of testing to combat the pandemic, has obvious policy implications. Second, the findings offered also imply the relevance of effectiveness of governments, which is negatively associated with deaths, though this relationship is only partially supported in the analysis. Regardless of regime types, the varying capacities of bureaucracy and administrations more generally seem to contribute to lowering the rates of casualties. This result is also consistent with Liang et al. (2020).

The analysis conducted in this paper, of course, has some limitations. For example, the yearly data used in the analysis allows only limited interpretations for the precise comparative assessments of the factors at work. Cepaluni et al. (2020) and Cheibub et al. (2020) utilize daily data, making a more nuanced analysis amenable for capturing daily fluctuations in the prevalence of COVID-19 as well as government interventions. As almost all other variables included in the analysis of this paper are yearly data, it is warranted to probe, in future research, how the data manipulation, regime characteristics, and government effectiveness, among other political variables, influence the pattern of pandemic containment in the shorter run or even on a daily basis.

Another limitation is the number of observations in the dataset. Unfortunately, the paper could only utilize a limited number of countries, mainly due to the missing values of Transparency Index, which is essential to this study. As noted earlier, some countries even do not report the number of death cases; we cannot judge from the data source whether this is zero or a missing value. Because these problems may lead to some bias, they should be detected and corrected in future research, though it would be an immensely difficult task.

## 6 Conclusion

A significant number of people have suffered from and died of COVID-19. The overwhelming number of casualties in democratic countries is daunting enough to make us wonder whether the very foundation of democracies, such as their respect for individual rights and freedom, may be liabilities in combatting the pandemic. Some scholars have indeed provided the view of “efficient autocracy,” with data showing the apparent correlations between regime types and public health in favor of nondemocratic countries. This view, however, remains unconvincing, and this study has attempted to challenge the validity of such a view. Authoritarian countries do not tend to have fewer COVID-19 deaths; instead, as suggested by this study, a critical determinant of the higher reported number of COVID-19 deaths is likely to be data transparency. To view the relationship between freedom and health as some sort of tradeoff is superficial and misleading. The current pandemic, though it often appears overwhelming, should not let us doubt the legitimacy of and the core values associated with democratic government.

## CRediT authorship contribution statement

**Susumu Annaka:** Conceptualization, Methodology, Formal analysis, Resources, Investigation, Software, Validation, Funding acquisition.

## Figures and Tables

**Table:** 

Variable	Obs	Mean	Std. Dev.	Min	Max
Confirmed Deaths per 1M pop	108	260.63	335.9418	0.08	1532
Confirmed Deaths per 1M pop (log)	108	4.2025	2.0844	-2.5257	7.3343
Polity2	108	5.0463	5.5254	-10	10
MPI	108	0.3766	0.2893	0	0.8410
Transparency Index	108	1.1747	1.9149	-1.5354	5.6357
Government Effectiveness	108	0.0668	0.9751	-2.0154	2.2211
Stringency Index	108	62.2557	12.8594	13.8177	91.5112
GDP per capita (log)	108	8.6967	1.5196	5.3509	11.4308
Population Density (log)	108	4.3559	1.3803	1.1780	8.9813
Age 65 and above Ratio (log)	108	1.9181	0.7705	0.0816	3.3170
Confirmed Cases 1M pop (log)	108	8.2713	1.9190	2.6391	10.8579
Number of Tests 1M pop (log)	108	11.0773	1.6203	7.6271	14.4242
Latitude	108	15.7294	25.5474	-40.9006	61.9241
Longitude	108	16.1894	62.8394	-102.5528	178.0650
Days since First Confirmed Case	108	286.8611	20.5442	213	325
Days since First Confirmed Case^2	108	82707.45	11901.6	45369	105625

**Table:** 

	Country name	
Afghanistan	Guatemala	Nigeria
Albania	Guinea	Norway
Angola	Guyana	Oman
Argentina	Haiti	Pakistan
Australia	Honduras	Panama
Austria	Hungary	Papua New Guinea
Bangladesh	India	Paraguay
Belgium	Indonesia	Peru
Benin	Iraq	Philippines
Bolivia	Ireland	Poland
Botswana	Israel	Portugal
Brazil	Italy	Romania
Bulgaria	Ivory Coast	Russia
Burundi	Jamaica	Rwanda
Cameroon	Japan	Saudi Arabia
Canada	Jordan	Senegal
Central African Republic	Kenya	Singapore
Chile	Korea South	South Africa
China	Kuwait	Spain
Colombia	Lebanon	Sri Lanka
Costa Rica	Lesotho	Swaziland
Cuba	Liberia	Sweden
Cyprus	Libya	Switzerland
Denmark	Madagascar	Thailand
Dominican Republic	Malawi	Togo
Ecuador	Malaysia	Trinidad and Tobago
Egypt	Mali	Tunisia
El Salvador	Mauritania	Turkey
Ethiopia	Mauritius	UAE
Fiji	Mexico	Uganda
Finland	Morocco	United Kingdom
France	Mozambique	United States
Gabon	Nepal	Uruguay
Gambia	Netherlands	Vietnam
Ghana	New Zealand	Zambia
Greece	Niger	Zimbabwe

**Table:** 

VARIABLES	(1)	(2)	(3)	(4)
	OLS	Poisson	OLS	Poisson
	Deaths cases per 1M (log)	Deaths cases per 1M	Deaths cases per 1M (log)	Deaths cases per 1M
Polity2	0.0282	0.0237		
	(0.0398)	(0.0265)		
MPI			1.084	1.060**
			(0.823)	(0.448)
**Transparency Index**	0.427***	0.292***	0.422***	0.315***
	**(0.100)**	**(0.0572)**	**(0.103)**	**(0.0626)**
Age 65 and above (ratio)	0.554	0.308	0.374	0.0481
	(0.353)	(0.202)	(0.405)	(0.245)
Constant	2.461***	4.138***	2.557***	4.315***
	(0.535)	(0.318)	(0.543)	(0.327)
Observations	121	121	122	122
Adjusted (or Pseudo) R-squared	0.3545	0.4597	0.3621	0.4827
AIC	474.6939	25773.33	476.2002	24830.77
BIC	485.8771	25784.51	487.4163	24841.99

Robust standard errors in parentheses.

*** p<0.01, ** p<0.05, * p<0.1.
